# Liposomal bupivacaine versus ropivacaine for surgical site infiltration in lumbar fusion: a prospective randomized controlled trial

**DOI:** 10.1080/07853890.2026.2704247

**Published:** 2026-07-26

**Authors:** Feng Yue, Wenwen Zhai, Yanhan Lyu, Qiang Qi, Min Li

**Affiliations:** ^a^ Department of Anesthesiology, Peking University Third Hospital, Beijing, China; ^b^ Department of Orthopedics, Peking University Third Hospital, Beijing, China

**Keywords:** Liposomal bupivacaine, spinal fusion, postoperative pain, analgesics, randomized controlled trial, ClinicalTrials.gov, NCT07171125, date: 2025-08-27

## Abstract

**Introduction:**

Effective postoperative pain control after lumbar spine surgery remains challenging, and excessive opioid use is associated with adverse outcomes. Evidence comparing liposomal bupivacaine (LB) with conventional long-acting local anesthetics in spine surgery is limited.

**Patients and Methods:**

In this single-center, prospective, randomized, patient- and outcome assessor-blinded trial, adult undergoing one- or two-level posterior lumbar decompression and fusion were assigned (1:1) to surgical site infiltration with either LB (266 mg) plus 25 mg plain bupivacaine (LB group) or ropivacaine (R group). The primary outcome was 72 h cumulative opioid consumption (morphine milligram equivalents, MME). Secondary outcomes included time-profile opioid consumption, pain scores, rescue analgesia, safety, and functional recovery.

**Results:**

A total of 202 patients were included in the modified intention-to-treat analysis. Cumulative MME within 72 h was significantly lower in the LB group compared with the R group [43.0 (37.0, 58.0) mg vs. 58.0 (46.0, 73.0) mg], corresponding to a 22% relative reduction (GMR 0.78, 95% CI 0.71–0.85; *p* < 0.001). The reduction was most pronounced during 8–24 h and 24–48 h postoperatively. Overall pain scores at rest and with movement, as well as 72-h pain AUC, were lower in the LB group. No significant between-group differences were observed in rescue analgesia, adverse events and functional recovery.

**Conclusion:**

In patients undergoing one- or two-level posterior lumbar decompression and fusion, surgical site infiltration with an LB-based combined regimen, compared with ropivacaine monotherapy, reduced 72-h opioid consumption and cumulative postoperative pain burden without an observed increase in adverse events or impairment of early functional recovery.

## KEY MESSAGES

1. In patients undergoing one- or two-level posterior lumbar decompression and fusion, surgical site infiltration with a liposomal bupivacaine-based combined infiltration regimen achieved a 22% relative reduction in 72-hour postoperative opioid consumption compared with ropivacaine.
2. Liposomal bupivacaine-based combined infiltration regimen significantly reduced the cumulative pain burden and worst pain intensity postoperatively, although no significant differences were observed in model-based pairwise comparisons at individual time points.
3. The opioid-sparing and analgesic benefits of the liposomal bupivacaine-based combined infiltration regimen were achieved without increasing the incidence of adverse events or compromising short-term functional recovery outcomes in lumbar fusion patients.

## 1. Introduction

Spinal surgery frequently induces severe postoperative pain due to extensive tissue injury involving vertebral structures, intervertebral discs, and neural elements, resulting in both nociceptive and neuropathic components [1,2]. Inadequate pain control may delay mobilization, increase the risk of complications such as deep vein thrombosis and pulmonary complications, and contribute to the development of chronic postsurgical pain [3–6].

Importantly, postoperative pain following spine surgery is not static but evolves dynamically, typically peaking within the first 24–48 h as a result of inflammatory response, muscle injury, and early mobilization demands. Therefore, effective analgesic strategies should not only reduce pain intensity but also align temporally with the evolving nociceptive burden.

Multimodal analgesia is the cornerstone of perioperative pain management in spine surgery [7]. Local anesthetic infiltration at the surgical site represents a key component of these strategies, as it directly targets the source of nociceptive input and facilitates early recovery [8,9]. Conventional regimens combining nonsteroidal anti-inflammatory drugs and opioids are widely used; however, their efficacy remains limited by short duration of action and opioid-related adverse effects [3].

Liposomal bupivacaine (LB) is an extended-release formulation that encapsulates bupivacaine within multivesicular lipid particles, enabling sustained drug release for up to 72 h [10–12]. Clinical studies across surgical specialties have suggested that LB may reduce postoperative pain and opioid consumption [13–18], and several relevant clinical trial protocols have also been initiated to further validate its analgesic efficacy in perioperative settings [19–21]. However, evidence in spine surgery remains inconsistent, and most studies are retrospective or methodologically heterogeneous [22–27]. Notably, the few available randomized controlled trials have compared LB with placebo rather than active long-acting local anesthetics [28,29], limiting their clinical applicability. Furthermore, previous studies have largely focused on static pain outcomes or cumulative opioid consumption without considering the temporal relationship between drug release profiles and postoperative pain trajectories. Whether an extended-release formulation such as LB confers clinical benefit through better temporal alignment with postoperative pain dynamics remains unclear.

Therefore, we conducted a prospective, randomized, patient- and outcome assessor-blinded trial to evaluate the effects of LB combined with plain bupivacaine versus ropivacaine for surgical site infiltration in patients undergoing one- or two-level posterior lumbar decompression and fusion. The primary objective was to assess opioid consumption within the first 72 h postoperatively, with secondary outcomes including pain trajectory, cumulative pain burden, and recovery-related endpoints. By incorporating time-dependent analyses, this study aimed to provide a more comprehensive evaluation of analgesic efficacy in the context of postoperative pain dynamics.

## 2. Patients and methods

This single-center, prospective, randomized, controlled, patient- and assessor-blinded clinical trial was carried out at Peking University Third Hospital. This trial was performed in accordance with the Declaration of Helsinki of the World Medical Association and approved by the hospital’s Institutional Review Board (IRB00006761-M20250040, date: 2025-09-02). Written informed consent was obtained from each participant after detailed clarification of the study aims, interventions, potential benefits and risks, and the right to withdraw from the study at any time without any adverse consequences. The safety and well-being of participants were the primary considerations throughout the study. The study was registered at ClinicalTrials.gov (NCT07171125, date: 2025-08-27) before patient recruitment. The trial was reported in accordance with the Consolidated Standards of Reporting Trials (CONSORT) guidelines. In accordance with the TITAN 2025 guideline, we confirmed that no artificial intelligence tools were used for study design, data collection, statistical analysis, or interpretation.

### 2.1. Subjects

Adult patients over 18 years old with American Society of Anesthesiologists (ASA) physical status class I–III, who were scheduled for elective one- or two-level posterior lumbar decompression and fusion with internal fixation for degenerative spinal diseases and planned to receive intravenous patient-controlled analgesia (PCA) after surgery, were eligible for recruitment.

The exclusion criteria were known allergy to any component of multimodal analgesia or local anesthetics, chronic pain requiring long-term opioid therapy, pre-existing neurological deficits interfering with pain assessment, active systemic or local infection, pregnancy or lactation, refusal to participate, and any other investigator-determined high-risk conditions.

### 2.2. Randomization and masking

Patients were enrolled randomly in a 1:1 ratio to receive ropivacaine only (R group) or liposomal bupivacaine combined with plain bupivacaine (LB group). A blocked randomization method with a block size of four was used, and randomization sequences were generated using the SAS statistical software package (Version 9.3, SAS Institute Inc., Cary, NC, USA). Random results were sealed in serially numbered opaque envelopes, which were kept by an independent trial coordinator. On the day of surgery, the envelope was opened after induction of general anesthesia, and study drugs were prepared accordingly.

The trial coordinator was not involved in anesthesia management, perioperative care, or postoperative follow-up. Due to the distinct milky appearance of LB suspension, complete blinding of the treating anesthesiologists and surgeons was not feasible. However, all patients, outcome assessors, and investigators responsible for data collection and follow-up were blinded to group assignments. Outcome assessors were not present during drug preparation or infiltration procedures.

### 2.3. Anesthesia, perioperative care, and intervention

During the preoperative visit on the day before surgery, patients received standardized training on the use of an 11-point numerical rating scale (NRS, 0 represented no pain, and 10 indicated the worst pain imaginable) and an intravenous PCA device. They were instructed to initiate PCA when pain reached NRS >3.

All patients received preemptive analgesia with NSAIDs (celecoxib or etoricoxib) on the morning of surgery. Intraoperative monitoring included blood pressure (BP), electrocardiogram, pulse oxygen saturation (SpO_2_), bispectral index (BIS), capnogram, inhalational anesthetic concentration, urine output, and temperature. Dexamethasone 5 mg was given pre-anesthesia to prevent postoperative nausea and vomiting. General anesthesia was induced using sufentanil, propofol, etomidate, and rocuronium. Anesthesia was maintained with sevoflurane inhalation and remifentanil infusion, targeting a BIS range between 40 and 60. Neuromuscular blockade was maintained with rocuronium as required. Fluid and transfusion management followed institutional protocols. Flurbiprofen 50 mg and ondansetron 4 mg were given 30 min before the end of surgery.

#### 2.3.1. Intervention protocols

The LB group received 266 mg LB combined with 25 mg plain bupivacaine. For single-level surgery: LB 266 mg (20 ml, undiluted) + plain bupivacaine 0.25% diluted to 10 ml; for two-level surgery: LB diluted to 30 ml + plain bupivacaine 0.25% diluted to 10 ml. These were administered bilaterally (LB and plain bupivacaine injected sequentially using separate syringes). The R group received 120 mg ropivacaine, diluted as 30 ml (0.4%) for single-level and 40 ml (0.3%) for two-level. All infiltrations were performed before wound closure using a standardized technique. A volume of 2–3 ml per injection site was delivered at 1.5–2 cm intervals along the incision, targeting both paraspinal muscles and subcutaneous tissues to ensure comprehensive coverage of the surgical field. Any immediate local anesthetic-related adverse reactions (e.g. systemic toxicity or hypersensitivity) were prospectively recorded.

Patients were extubated in the operating room, then monitored in the Post-Anesthesia Care Unit (PACU) for at least 30 min. Intravenous sufentanil 5 μg was used as rescue analgesia if NRS ≥ 4. All patients received an intravenous morphine PCA pump for 72 h (1 mg bolus, with a 10-min lockout, no background infusion, and a maximum of 15 mg/5 h). If pain persisted (NRS ≥ 4 after three boluses), tramadol 50–100 mg was given as rescue therapy. Flurbiprofen 50 mg was administered intravenously twice daily for two days postoperatively.

### 2.4. Data collection and measurements

Baseline data of patient characteristics, diagnosis, and preoperative pain scores were recorded. Intraoperative data included spinal levels, durations of surgery, anesthesia medications, fluid infusion, blood loss, and transfusion.

#### 2.4.1. Pain-related outcomes

The primary outcome was cumulative opioid consumption expressed as morphine milligram equivalents (MME) within 72 h after surgery. Patients were assessed at PACU discharge and at 4, 8, 24, 48, and 72 h (±30 min window). Assessments scheduled between 21:00 and 7:00 were deferred to 08:00 to avoid sleep disruption.

Pain (rest and movement), rescue analgesics, and sleep disturbance were recorded. Sleep disturbance due to pain was recorded as a binary variable (yes/no), defined as any self-reported awakening or difficulty initiating sleep attributable to pain during the postoperative nights. We also recorded time to first PCA attempt, worst postoperative NRS pain score, and patient pain management satisfaction score (0–10, with higher scores indicating greater satisfaction).

#### 2.4.2. Adverse events and postoperative complications

During the postoperative days 1 to 3, systematic observation was conducted to evaluate local wound complications (including hematoma and surgical site infection) and opioid-related adverse events, including desaturation (SpO_2_<90% on room air), deep sedation (Richmond Agitation–Sedation Scale≤-3), 3D-CAM (3-Minute Diagnostic Interview for CAM-Defined Delirium) confirmed delirium, nausea/vomiting, pruritus, and delayed urinary catheter removal (>24 h postoperatively).

#### 2.4.3. Functional recovery assessment

Postoperative functional recovery included time to first ambulation, time to first oral intake, bowel movement, urinary catheter removal, and hospital stay.

### 2.5. Statistical analysis

The primary outcome measure was the total opioid consumption within the first 72 postoperative hours. At 72 h after surgery, all opioid analgesics administered *via* PCA, IV, or oral (PO) routes were converted into morphine milligram equivalents (MME) using standard conversion factors.

Based on retrospective pilot data from our institution, the sample size estimation assumed a mean cumulative 72-h opioid consumption of 72 mg MME and a standard deviation of 45 mg. In the absence of an established minimum clinically important difference (MCID), a 30% relative reduction was prespecified as the clinically meaningful effect size for this trial. With a two-sided *α* of 0.05 and power of 90%, 92 patients per group were required. Allowing for a 10% dropout rate, the final planned sample size was set at 102 patients in each group.

Baseline balance between groups was evaluated using the absolute standardized difference (ASD). ASD for continuous variables was calculated as the absolute difference in group means divided by the pooled standard deviation, while for binary variables, it was determined as the absolute difference in proportions divided by the pooled standard deviation of the proportions. An ASD threshold of 0.276 was used to indicate meaningful imbalance, based on the formula $1.96\times \sqrt{\left(n1+n2)/(n1\times n2\right)}$, with *n*_1_ = *n*_2_ = 101.

Categorical variables were displayed as counts (percent) and assessed using Pearson’s chi-square test or Fisher’s exact test, as appropriate. Continuous variables were checked for normality *via* the Kolmogorov-Smirnov test. Normally distributed variables were shown as mean ± standard deviation (SD) and compared using independent samples t-tests, with effect estimates reported as mean differences (MD) and 95% confidence intervals (CIs). Non-normally distributed variables were presented as medians (interquartile range, IQR) and analyzed with the Mann–Whitney U test or Wilcoxon rank-sum test; Hodges–Lehmann median differences and 95% CIs were estimated for between-group differences. For binary outcomes, relative risks (RRs) and 95% CIs were calculated. The time‑weighted area under the curve (AUC) of NRS pain scores over 72 h was used to evaluate overall pain.

For the primary outcome, cumulative opioid consumption over 72 h postoperatively was evaluated using a log-transformed linear regression model to account for skewed distribution, with results reported as geometric mean ratios (GMRs) with 95% CIs. Postoperative opioid consumption across predefined time intervals (0–4 h, 4–8 h, 8–24 h, 24–48 h, and 48–72 h) was analyzed through a log‑transformed repeated‑measures mixed‑effects linear regression model, including group, time, and group-by-time interaction as fixed effects, and patient as a random intercept.

Pain scores at rest and during movement were analyzed as repeated continuous outcomes using repeated-measures mixed-effects linear regression models with the same fixed- and random-effects structure. For repeated NRS pain outcomes, values were log-transformed after adding 0.1 to accommodate zero values. Model assumptions were assessed graphically using residual plots and normal Q–Q plots. Overall treatment effects from the primary model were back-transformed and reported as GMRs with 95% CIs. Because NRS scores are bounded ordinal variables, an untransformed linear mixed-effects model with the same fixed- and random-effects structure was performed as a sensitivity analysis to facilitate interpretation on the original NRS scale. Model-based marginal means were estimated from this untransformed model. Time-point-specific pairwise between-group comparisons from this untransformed model were Bonferroni-adjusted and treated as supportive analyses. Raw median NRS scores were presented descriptively without pointwise inferential testing. Time to first PCA attempt was analyzed using log-transformed regression models and reported as GMRs.

Subgroup analyses were prespecified and considered exploratory and were conducted using the same statistical approaches as the primary analysis, with interaction terms used to assess heterogeneity of treatment effects. Efficacy analyses were primarily performed on a modified intention-to-treat (mITT) basis, with per-protocol (PP) analyses conducted as sensitivity analyses for the primary outcome. For the mITT analysis, 72-h cumulative MME was derived from recorded PCA use, documented non-PCA opioid administration, and available verifiable PCA-related records. When PCA records were incomplete or PCA was discontinued before 72 h, cumulative opioid consumption was calculated using available recorded PCA consumption, verifiable PCA-related records when available (e.g. residual pump volume at pump removal), and any subsequently documented opioid administration through 72 h. No statistical imputation was performed. Patients with incomplete PCA records or PCA duration shorter than 72 h were excluded from the PP analysis. A two-sided *p* < 0.05 was considered statistically significant. SPSS Statistics 27.0 (IBM Corp., Armonk, NY, USA) was used for all analyses.

## 3. Results

### 3.1. Baseline characteristics and treatment

**Figure 1.**
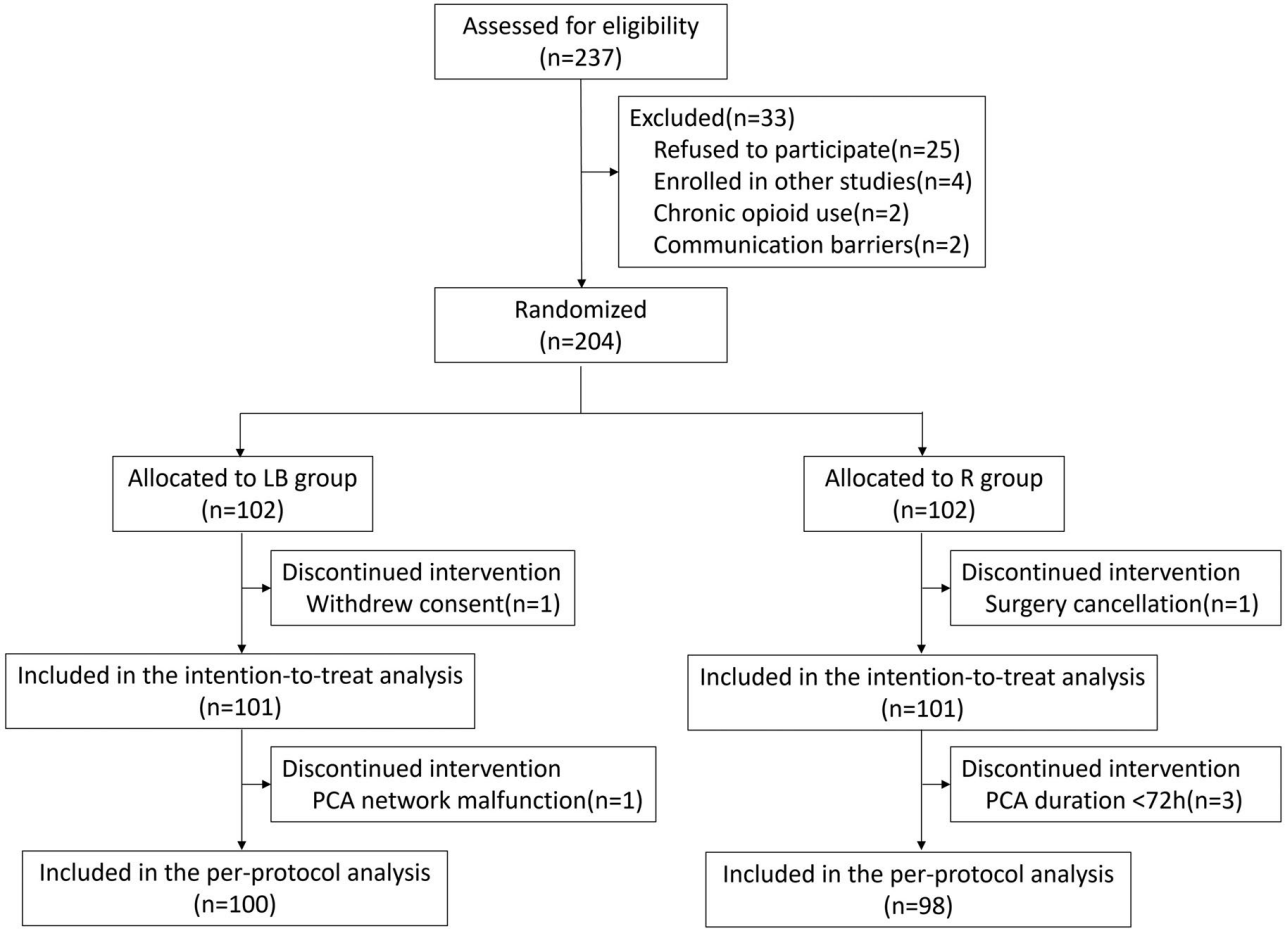
Patient flow diagram. LB, liposomal bupivacaine; R, ropivacaine. PCA, patient-controlled analgesia.

From September 20, 2025, to February 25, 2026, a total of 237 patients were assessed for eligibility, of whom 33 were excluded. A total of 204 patients were randomly assigned to the R group (*n* = 102) or LB group (*n* = 102). During the study period, one patient in the R group was excluded due to a severe allergic reaction to prophylactic antibiotics prior to anesthesia induction, leading to cancellation of surgery. One patient in the LB group withdrew consent before anesthesia. Four protocol deviations were noted: one patient had incomplete PCA data due to device malfunction after 24 h, and three patients had PCA durations shorter than 72 h. Consequently, the mITT analysis included 202 patients, and the PP analysis included 198 participants (Figure 1).

The baseline demographic and clinical characteristics were well balanced between groups, with no meaningful imbalance observed. Intraoperative variables, including durations of surgery, number of spinal levels, intraoperative opioid use, fluid administration, urine output, blood loss, and transfusion, were comparable between groups (Table 1).

**Table 1.** Patient characteristics and intraoperative data.

Characteristics	LB group (*n* = 101)	R group (*n* = 101)	ASD^a^
Age (yr)	62 (56, 69)	60 (54, 67)	0.162
Sex, female, *n* (%)	63 (62.4)	64 (63.4)	0.021
Weight (kg)	70.5 ± 11.7	71.8 ± 11.9	0.115
Height (cm)	164.5 ± 8.0	165.2 ± 7.7	0.090
BMI (kg/m^2^)	26.0 ± 3.5	26.2 ± 3.3	0.073
ASA physical status, *n* (%)			0.200
1	13 (12.9)	7 (6.9)	
2	82 (81.2)	88 (87.1)	
3	6 (5.9)	6 (5.9)	
Preoperative NRS of pain, at rest	2 (1, 2)	2 (0, 2)	0.134
Preoperative NRS of pain, with movement	2 (1, 3)	2 (0, 3)	0.239
Preoperative analgesics, *n* (%)^b^	29 (28.7)	19 (18.8)	0.233
Preoperative hypnotic use, *n* (%)^c^	5 (5.0)	1 (1.0)	0.233
Duration of surgery (min)	148.9 ± 40.6	139.8 ± 43.3	0.217
Number of operated spinal levels, *n* (%)			0.086
1	61 (60.4)	64 (63.4)	
2	40 (39.6)	37 (36.6)	
Sufentanil (μg)	15 (15, 20)	15 (15, 20)	0.228
Remifentanil (μg)	826 (665, 1030)	706 (582, 1006)	0.218
Total fluid infusion (ml)	1800 (1488, 2220)	1800 (1330, 1872)	0.262
Crystalloid (ml)	1200 (1100, 1500)	1200 (1100, 1400)	0.067
Colloid (ml)	500 (0, 500)	500 (0, 500)	0.230
Autologous blood (ml)	120 (100, 180)	125 (110, 156)	0.045
Allogeneic blood (ml)	0 (0, 0)	0 (0, 0)	0.262
Estimated blood loss (ml)	300 (200, 400)	300 (200, 400)	0.044
Urine output (ml)	500 (300, 700)	400 (300, 600)	0.200

Data are mean ± standard deviation, median (IQR), or *n* (%).

^a^ASD, absolute standardized difference ≥ 0.276 were considered indicative of imbalance between groups.

^b^Number and percentage of patients with daily analgesic use.

^c^Number and percentage of patients with regular hypnotic use.

LB, liposomal bupivacaine; R, ropivacaine; BMI, body mass index; ASA, American Society of Anesthesiologists; NRS, numerical rating scale.

### 3.2. Primary outcome

Cumulative opioid consumption (converted to MME) within the first 72 h postoperatively was significantly lower in the LB group than in the R group [43.0 (37.0, 58.0) mg vs. 58.0 (46.0, 73.0) mg], indicating a relative reduction of approximately 22% in opioid consumption (GMR 0.78, 95% CI 0.71–0.85; *p* < 0.001). This finding was consistent in the PP analysis (GMR 0.77, 95% CI 0.70–0.84; *p* < 0.001) (Table 2).

**Table 2.** Postoperative opioid consumption and pain control.

	LB group (*n* = 101)	R group (*n* = 101)	Estimated effect (95% CI)^a^	*P*
Postoperative MME within 72h (mg) (mITT analysis)	43.0 (37.0, 58.0)	58.0 (46.0, 73.0)	GMR 0.78 (0.71–0.85)	<0.001
Postoperative MME within 72h (mg) (PP analysis)	43.0 (37.0, 58.0) (*n* = 100)	58.0 (46.5, 73.0) (*n* = 98)	GMR 0.77 (0.70–0.84)	<0.001
Interval opioid consumption (mg)			GMR 0.81 (0.73–0.89)	<0.001
0–4 h	1.5 (1.5, 4.5)	1.5 (1.5, 7.0)	GMR 0.86 (0.71–1.03)	0.103
4–8 h	1.5 (1.5, 4.5)	1.5 (1.5, 4.5)	GMR 0.86 (0.72–1.03)	0.105
8–24 h	10.0 (7.0, 10.0)	13.0 (10.0, 16.0)	GMR 0.70 (0.58–0.83)	<0.001
24–48 h	13.5 (10.5, 16.5)	16.5 (13.5, 25.5)	GMR 0.70 (0.58–0.84)	<0.001
48–72 h	13.5 (10.5, 19.5)	16.5 (10.5, 22.5)	GMR 0.96 (0.76–1.22)	0.668
AUC of NRS-R_0-72h_^b^	65.1 ± 53.7	85.0 ± 63.0	MD −19.9 (−36.1– −3.7)	0.017
AUC of NRS-M_0-72h_^c^	186.3 ± 78.1	225.1 ± 79.2	MD −38.8 (−60.6– −17.0)	<0.001
Patients requiring sufentanil in PACU	6 (5.9)	13 (12.9)	RR 0.46 (0.19–1.11)	0.088
Patients requiring rescue tramadol in ward	2 (2.0)	6 (5.9)	RR 0.33 (0.07–1.59)	0.144
Sleep disturbance due to pain in 3-day period	7 (6.9)	11 (10.9)	RR 0.64 (0.26–1.54)	0.323
Time to first PCA attempt (h)	4 (3, 21)	8 (4, 22)	GMR 0.85 (0.61–1.19)	0.334
Worst NRS	4 (4, 5)	5 (4, 6)	HL −1.0 (−1.0–0.0)^d^	0.001
Patient pain management satisfaction score	10 (10, 10)	10 (10, 10)	HL 0.0 (0.0–0.0)	0.175^e^

Data are mean ± standard deviation, median (IQR), or *n* (%).

^a^Estimated effects were presented as geometric mean ratios (GMRs) for opioid consumption and time-related outcomes (derived from log-transformed models), mean differences for AUC pain scores, Hodges–Lehmann median difference for worst NRS pain score and patient satisfaction score, risk ratios for binary outcomes. For GMR and RR, effects are expressed as the LB group versus the R group. For mean differences and Hodges–Lehmann median difference, effects are expressed as the LB group minus the R group.

^b^Area under the curve of NRS score at rest 72 h postoperatively.

^c^Area under the curve of NRS score with movement 72 h postoperatively.

^d^Worst postoperative NRS pain score was compared using the Wilcoxon rank-sum test, with the effect size expressed as the Hodges–Lehmann median difference. Because NRS is a discrete ordinal variable, the rounded confidence interval may display an upper bound of 0.0 despite a statistically significant rank-sum test. With greater precision, the upper bound of the 95% confidence interval was slightly below 0 (−0.00002).

^e^For discrete outcomes with many ties, Hodges–Lehmann confidence intervals may appear as 0.0–0.0 after rounding despite non-identical rank distributions; *P* values were derived from Mann–Whitney *U* tests.

LB, liposomal bupivacaine; R, ropivacaine; MME, morphine milligram equivalent; mITT, modified intention-to-treat; PP, per-protocol; CI, confidence interval; GMR, ratio of geometric means; RR, risk ratio; AUC, area under the curve; PACU, post-anesthesia care unit; PCA, patient-controlled analgesia; NRS, numerical rating scale; HL, Hodges–Lehmann median difference.

### 3.3. Secondary outcomes

Analysis using a log-transformed repeated-measures mixed-effects model demonstrated a significant overall reduction in opioid consumption in the LB group (GMR for LB vs. R 0.81, 95% CI 0.73–0.89; *p* < 0.001) with a significant group-by-time interaction (*p* = 0.027). Specifically, opioid requirements were significantly lower in the LB group during the 8–24 h (GMR 0.70, 95% CI 0.58–0.83; *p* < 0.001) and 24–48 h (GMR 0.70, 95% CI 0.58–0.84; *p* < 0.001) intervals. No significant differences were observed during 0–4 h, 4–8 h, or 48–72 h (Table 2).

Repeated-measures analysis of log-transformed NRS scores showed that the LB group had significantly lower overall pain scores compared with the ropivacaine group at both rest (GMR 0.80, 95% CI 0.68–0.94; *p* = 0.007) and during movement (GMR 0.84, 95% CI 0.75–0.93; *p* = 0.001). Sensitivity analyses using an untransformed linear mixed-effects model yielded consistent results on the original NRS scale for both pain at rest and pain with movement (overall pain at rest: MD −0.253, 95% CI −0.461 to −0.046; pain with movement: MD −0.442, 95% CI −0.729 to −0.155).

**Figure 2.**
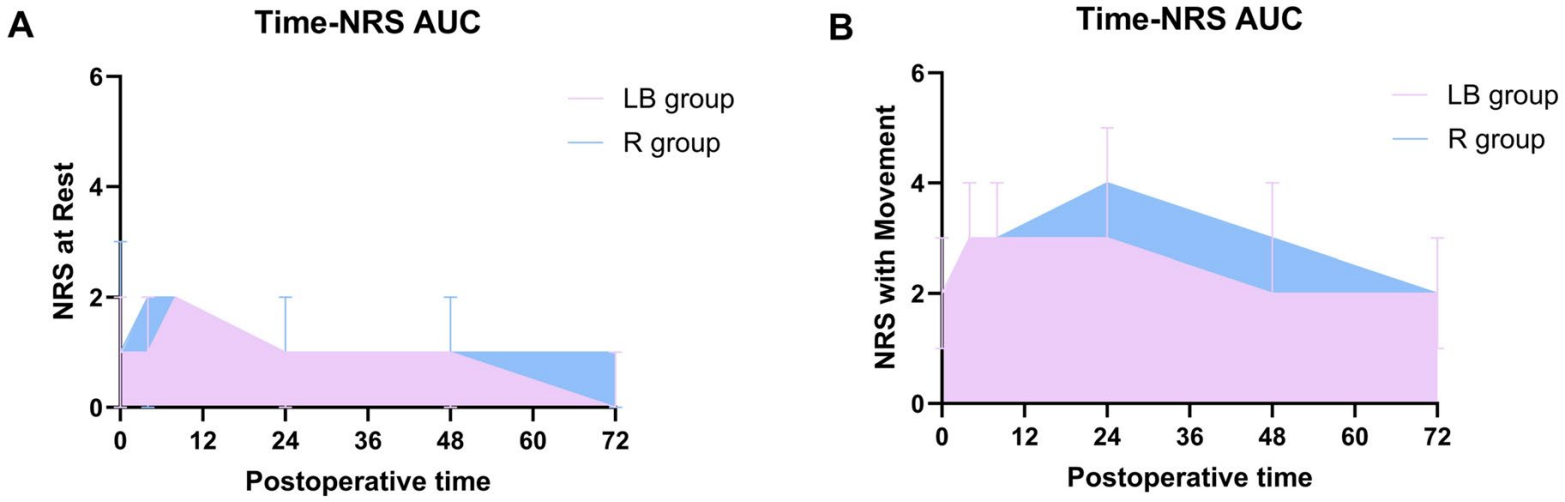
Comparison of the cumulative AUC of the NRS score at rest (A) and with movement (B) 72 h postoperatively. Data are plotted as mean ± standard deviation. LB, liposomal bupivacaine; R, ropivacaine; AUC, area under the curve; NRS, numerical rating scale.

Consistently, the time-weighted AUC of NRS scores over 72 h was significantly lower in LB group for both rest (MD −19.9, 95% CI −36.1– −3.7; *p* = 0.017) and movement-related pain (MD −38.8, 95% CI −60.6– −17.0; *p* < 0.001) (Table 2, Figure 2).

**Figure 3.**
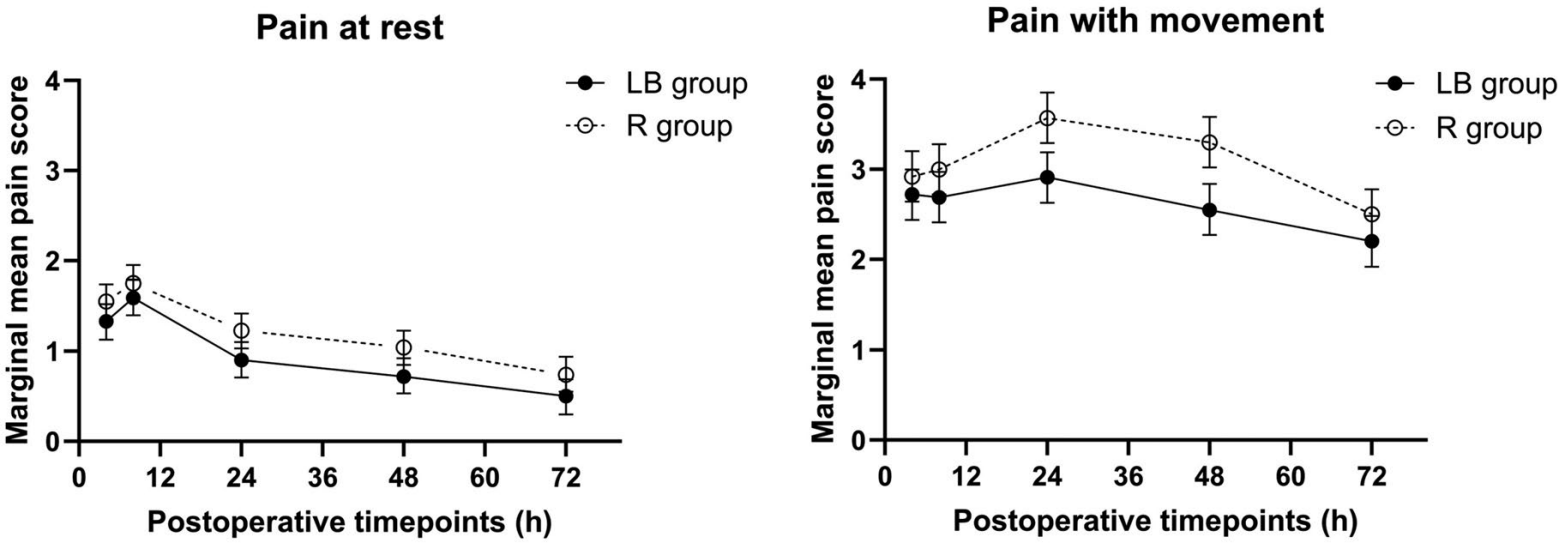
Marginal mean numerical rating scale pain scores at different time points. Data are expressed as marginal means with 95% confidence intervals estimated from the untransformed repeated-measures mixed-effects model. LB, liposomal bupivacaine; R, ropivacaine.

Model-based marginal mean NRS scores estimated from the untransformed mixed-effects model were presented in Figure 3 and Supplementary Table A for clinical-scale interpretation. These Bonferroni-adjusted time-point-specific comparisons were considered supportive analyses, and none reached statistical significance. Raw median NRS scores by time point were provided descriptively in Supplementary Table B and were not used for pointwise inferential conclusions.

The proportion of patients requiring rescue sufentanil in the PACU or tramadol in the ward did not differ significantly between groups. Time to first PCA attempt was comparable between groups. The LB group had a lower worst postoperative NRS pain score [4 (4,5) vs. 5 (4,6), Hodges–Lehmann median difference of −1.0, 95% CI −1.0 to 0.0; *p* = 0.001]. The incidence of pain-related sleep disturbance did not differ significantly between groups (Table 2).

The incidence of adverse events was comparable between groups. No local anesthetic-related or wound-related complications were observed in either group, and no cases of desaturation or deep sedation occurred. Functional recovery outcomes were also similar between groups, with no statistically significant differences detected (Table 3).

**Table 3.** Safety outcomes and functional recovery assessment.

	LB group (*n* = 101)	R group (*n* = 101)	Estimated effect (95% CI)^a^	*P*
Local anesthetic-related complications^b^	0	0	Not estimable	–
Wound-related complications^c^	0	0	Not estimable	–
Opioid-related adverse events				
Desaturation^d^	0	0	Not estimable	–
Delirium within 3 days	1 (1.0)	1 (1.0)	RR 1.00 (0.06–15.89)	>0.999^f^
Deep sedation^e^	0	0	Not estimable	–
Nausea or vomiting	10 (9.9)	12 (11.9)	RR 0.83 (0.38–1.84)	0.684
Pruritus	0	2 (2.0)	Not estimable	0.498^f^
Urinary catheter removal beyond 24 h postoperatively	13 (12.9)	16 (15.8)	RR 0.81 (0.41–1.60)	0.544
Time to first ambulation (h)	24.0 (20.0, 28.0)	24.0 (19.0, 31.0)	HL 1.0 (−1.0–3.0)	0.456
Time to first tolerated oral intake (h)	4.0 (2.0, 6.0)	2.0 (2.0, 6.0)	HL 0.0 (0.0–1.0)	0.147
Time to first bowel movement (h)	10.0 (7.0, 12.0)	10.0 (6.0, 15.0)	HL 0.0 (−2.0–1.0)	0.780
Time to successful removal of urinary catheter (h)	6.0 (4.0, 16.0)	6.0 (4.0, 16.0)	HL 0.0 (0.0–0.0)	0.187
Length of hospital stay after surgery (d)	5.0 (5.0, 5.0)	5.0 (5.0, 5.0)	HL 0.0 (0.0–0.0)	0.335
Length of hospital stay (d)	8.0 (7.0, 8.0)	8.0 (7.0, 8.0)	HL 0.0 (0.0–0.0)	0.457

Data are *n* (%) or median (IQR).

^a^For binary outcomes, relative risks were calculated as the LB group versus the R group. For continuous recovery outcomes, estimated effects are Hodges–Lehmann median difference with 95% confidence intervals, calculated as the LB group minus the R group.

^b^Local anesthetic systemic toxicity and allergic reactions.

^c^Hematoma and surgical site infection.

^d^Pulse oxygen saturation (breathing air) <90%.

^e^Richmond Agitation Sedation Scale≤-3.

^f^*P* value for Fisher exact test.

For discrete outcomes with many ties, Hodges–Lehmann confidence intervals may appear as 0.0–0.0 after rounding despite non-identical rank distributions; *P* values were derived from Mann–Whitney *U* tests.

LB, liposomal bupivacaine; R, ropivacaine; CI, confidence interval; RR, risk ratio; HL, Hodges–Lehmann median difference.

### 3.4. Subgroup analyses

Prespecified subgroup analyses were exploratory and should be interpreted cautiously because the study was not powered for subgroup comparisons. The opioid-sparing effect of LB was consistently observed across age, sex, BMI, and number of operated spinal levels subgroups. The subgroup-specific GMRs ranged from 0.75 to 0.80, and all interaction *P* values were >0.05. These findings may suggest that the primary opioid-sparing effect was generally consistent across clinically relevant subgroups (Table 4).

**Table 4.** Subgroup analysis on cumulative opioid consumption during the initial 72 h postoperatively.

Subgroup	No. of patients	LB group MME	R group MME	GMR (95% CI)^a^	*P*	*P* for interaction
Overall	202	43.0 (37.0, 58.0)	58.0 (46.0, 73.0)	0.78 (0.71–0.85)	<0.001	
Age, years						0.751
<55	49	43.0 (37.0, 70.0)	62.5 (49.0, 73.0)	0.80 (0.66–0.97)	0.026	
≥55	153	43.0 (34.0, 55.5)	58.0 (46.0, 73.0)	0.78 (0.70–0.86)	<0.001	
Sex						0.530
Male	75	40.0 (34.5, 58.0)	61.0 (46.0, 80.5)	0.75 (0.63–0.89)	0.001	
Female	127	43.0 (37.0, 55.0)	58.0 (46.0, 73.0)	0.80 (0.71–0.89)	<0.001	
BMI, kg/m^2^						0.914
<25	73	43.0 (37.0, 53.5)	55.0 (46.0, 73.0)	0.79 (0.69–0.90)	<0.001	
≥25	129	43.0 (34.0, 58.0)	58.0 (46.0, 73.5)	0.78 (0.69–0.88)	<0.001	
Number of operated spinal levels						0.594
1	125	43.0 (34.0, 58.0)	58.0 (46.0, 73.0)	0.79 (0.71–0.89)	<0.001	
2	77	43.0 (37.0, 50.5)	58.0 (46.0, 76.0)	0.75 (0.64–0.89)	<0.001	

Data are median (IQR). GMRs, 95% CIs, and P values were estimated using log-linear regression models within each subgroup, with log-transformed 72-hour cumulative opioid consumption as the dependent variable and treatment group as the independent variable. *P* values for interaction were derived from models including treatment group, subgroup, and treatment-by-subgroup interaction terms.

^a^For GMR: LB group vs. R group.

LB, liposomal bupivacaine; R, ropivacaine; CI, confidence interval; GMR, geometric mean ratio; BMI, body mass index.

## 4. Discussion

In this prospective randomized trial, LB-based combined wound infiltration regimen resulted in a significant reduction in cumulative opioid consumption over the first 72 h postoperatively compared with ropivacaine, with consistent findings in both modified intention-to-treat and per-protocol analyses. The magnitude of opioid reduction (approximately 20–25%) might be clinically relevant in the context of contemporary efforts to minimize perioperative opioid exposure, particularly in spine surgery, where postoperative pain was often severe and prolonged. The present findings should be interpreted in the context of the study intervention. The LB group received LB combined with plain bupivacaine to provide early analgesic coverage before the sustained-release phase of LB, whereas the control group received ropivacaine alone. Therefore, the observed opioid-sparing and analgesic effects reflect the efficacy of an LB-based combined infiltration regimen, rather than the effect of LB monotherapy.

This finding was further supported by the time-dependent analysis, which demonstrated that the opioid-sparing effect of LB was most pronounced during the 8–48 h postoperative period. This temporal pattern aligned closely with the known trajectory of postoperative pain following lumbar spine surgery, characterized by peak nociceptive input due to muscle dissection, inflammatory response, and early mobilization demands during this phase [8]. The observed analgesic benefit was consistent with the pharmacokinetic profile of liposomal bupivacaine, which provided sustained drug release through multivesicular liposomal encapsulation [12,30]. Importantly, our findings extended beyond a simple duration effect and suggested a time-matched analgesic advantage, whereby prolonged local anesthetic availability overlapped with the peak phase of postoperative nociceptive input. This temporal alignment might explain why the between-group differences were most evident at 8–48 h, but not during the early (0–8 h) or late (48–72 h) postoperative periods.

Moreover, the lack of early analgesic superiority supported the notion that liposomal bupivacaine alone may not provide sufficient immediate postoperative analgesia due to its relatively slow initial release kinetics [31], as previously suggested in pharmacodynamic studies [32,33]. The addition of plain bupivacaine in our protocol likely compensated for this limitation, providing early analgesic coverage and enabling a smoother transition to the sustained-release phase.

The clinical relevance of the observed opioid reduction should be interpreted in both relative and absolute terms and in relation to pain-related patient-centered outcomes. The 22% relative reduction corresponded to a 15 mg median reduction in MME over 72 h and was accompanied by lower cumulative pain burden and a 1-point reduction in the worst postoperative NRS pain score. Together, these findings may represent a modest but patient-relevant opioid-sparing and analgesic benefit.

In addition to opioid reduction, LB was associated with lower overall pain burden, as reflected by reduced pain scores and lower worst pain intensity. These findings were clinically relevant because cumulative pain exposure and peak pain intensity had both been linked to poorer recovery trajectories and increased risk of persistent postsurgical pain [34,35].

Interestingly, while the overall pain trajectory differed between groups, pairwise comparisons at individual time points did not reach statistical significance, suggesting that conventional time-point analyses might underestimate treatment effects in postoperative pain studies. In this context, the use of repeated-measures modeling and time-integrated measures such as AUC provided a more comprehensive assessment of analgesic efficacy over time.

Our findings were broadly consistent with prior studies demonstrating opioid-sparing effects of liposomal bupivacaine in spine surgery [22–26,36]. However, previous literature had reported heterogeneous results, with several studies failing to demonstrate significant benefits [29,37–39]. Such discrepancies were likely attributable to differences in study design, drug administration strategies, and infiltration techniques rather than an absence of treatment effect. First, studies using liposomal bupivacaine alone may have underestimated its clinical benefit due to insufficient early analgesic coverage [29,38,39]. Second, variability in infiltration techniques (e.g. paraspinal muscle-only or subcutaneous-only injection [37]) might reduce analgesic efficacy. Our standardized dual-plane infiltration strategy targeting both paraspinal muscle and subcutaneous tissue likely enhanced nociceptive blockade across the entire surgical field, which might partially explain the observed treatment effect.

Randomized evidence on LB in adult spine surgery remains limited, particularly in lumbar fusion trials using an active long-acting local anesthetic comparator. A recent meta-analysis by Daher et al. [17] reported potential opioid-sparing and analgesic benefits of LB across mixed spine procedures, although heterogeneity in surgical populations and analgesic protocols was substantial. The present trial adds active-comparator randomized evidence by comparing an LB-based combined infiltration regimen with ropivacaine in patients undergoing lumbar fusion.

Subgroup analyses demonstrated a consistent opioid-sparing effect of LB across age, sex, BMI, and surgical level categories, with no significant interactions observed. The subgroup analyses were exploratory and hypothesis-generating, as the study was not powered to detect subgroup-specific treatment effects or treatment-effect heterogeneity.

Adverse events were infrequent and comparable between groups, with no observed local anesthetic-related or wound-related complications. However, the low incidence of events limits the ability to draw firm conclusions regarding safety differences. Despite reduced opioid consumption and lower pain burden in the LB group, short-term functional recovery outcomes were similar between groups. This may be partly explained by the standardized ERAS pathway [40–42], in which milestones such as ambulation, oral intake, catheter removal, and discharge were influenced by protocolized care and surgical factors in addition to pain control. Once pain was sufficiently controlled to allow mobilization, further analgesic improvement may not have translated into earlier achievement of these early recovery milestones. Longer-term outcomes, such as persistent opioid use, chronic postsurgical pain, and return to work, were not assessed and warrant further investigation.

Cost is an important consideration for the clinical implementation of LB-based infiltration. Although the domestic LB formulation used in our setting may have a lower acquisition cost than imported formulations used in many previous studies, it remains more expensive than conventional local anesthetics such as ropivacaine. In the present trial, LB-based infiltration produced modest opioid-sparing and analgesic benefits, but we did not collect cost, nursing workload, rescue-treatment burden, or other resource-utilization outcomes. Therefore, no conclusion can be drawn regarding cost-effectiveness. Whether the additional drug cost is justified should be evaluated in future health-economic analyses that incorporate local drug prices and perioperative resource use.

This study had several limitations. First, treating anesthesiologists and operating surgeons could not be fully blinded because of the characteristic appearance of the LB suspension, which may have introduced performance bias. To minimize this risk, anesthesia management, infiltration procedures, and postoperative multimodal analgesia were standardized, and patients and outcome assessors remained blinded. Nevertheless, residual performance bias could not be entirely excluded. Second, the single-center design may limit generalizability of results to other clinical settings with different patient populations or perioperative protocols. Third, long-term outcomes (e.g. chronic pain development, opioid use, and functional recovery beyond discharge) were not assessed, which limited the ability to evaluate the sustained clinical impact of LB.

Despite these limitations, this study provided prospective randomized evidence supporting the potential role of an LB-based combined wound infiltration regimen in lumbar spine surgery. The demonstrated opioid-sparing effect and improved pain control were clinically relevant in the context of ongoing efforts to reduce perioperative opioid exposure and optimize ERAS pathways [42].

## 5. Conclusion

In patients undergoing one- or two-level posterior lumbar decompression and fusion, surgical site infiltration with an LB-based combined regimen, compared with ropivacaine monotherapy, reduced 72-h opioid consumption and cumulative postoperative pain burden without an observed increase in adverse events or impairment of early functional recovery. These findings support the role of an LB-based combined wound infiltration regimen as a component of multimodal analgesia in spinal surgery, although its impact on longer-term recovery outcomes requires further investigation.

## Supplementary Material

CONSORT 2025 checklist.pdf

Supplementary_Table_B.docx

Supplementary Table A.docx

## Data Availability

The raw data and materials used and/or analyzed during the current study will be available upon reasonable request from the corresponding author (liminanesth@bjmu.edu.cn).

## Research registration unique identifying number (UIN)

This study was registered on ClinicalTrials.gov (unique number NCT07171125), date: 2025-08-27.
