# Supplementary material for: Liposomal bupivacaine versus ropivacaine for surgical site infiltration in lumbar fusion: a prospective randomized controlled trial
Source: Ann Med. 2026 Jul 26;58(1):2704247. doi: 10.1080/07853890.2026.2704247 (PMC13410536; doi:10.1080/07853890.2026.2704247)
Supplement: CONSORT 2025 checklist.pdf [file IANN_A_2704247_SM4503.pdf]

CONSORT 2025 Checklist – Reported Page Numbers

Manuscript: Liposomal Bupivacaine Versus Ropivacaine in Lumbar Fusion

**Section/topic**	**No**	**CONSORT 2025 checklist item**	**Reported on page no.**
Title and abstract	1a	Identification as a randomised trial	p.1, 2, 6
Title and abstract	1b	Structured summary of the trial design, methods, results, and conclusions	p.2-3
Open science	2	Trial registration (registry, ID, URL, date)	p.7 and p.20
Open science	3	Where protocol and statistical analysis plan can be accessed	p.21
Open science	4	Data sharing (where/how data, code, materials can be accessed)	p.21
Funding and conflicts of interest	5a	Sources of funding/role of funders	p.20
Funding and conflicts of interest	5b	Financial and other conflicts of interest	p.20
Introduction	6	Scientific background and rationale	p.5-6
Introduction	7	Specific objectives related to benefits and harms	p.6
Methods	8	Patient/public involvement	None
Methods	9	Trial design (type, allocation ratio, framework)	p.6-7
Methods	10	Important protocol changes after commencement, with reasons	None
Methods	11	Settings and locations	p.6-7
Methods	12a	Eligibility criteria for participants	p.7
Methods	12b	Eligibility criteria for sites/providers (if applicable)	Not applicable (single-centre)
Methods	13	Intervention and comparator with replication details	p.9

Methods	14	Prespecified primary and secondary outcomes	p.10-11
Methods	15	How harms were defined and assessed	p.10-11
Methods	16a	Sample size determination and assumptions	p.11
Methods	16b	Interim analyses and stopping guidelines	None
Methods	17a	Sequence generation (who and method)	p.7
Methods	17b	Type of randomisation and restrictions	p.7
Methods	18	Allocation concealment mechanism	p.7
Methods	19	Implementation (who enrolled/assigned; access to sequence)	p.7
Methods	20a	Blinding: who was blinded	p.8
Methods	20b	How blinding was achieved; similarity of interventions	p.8
Methods	21a	Statistical methods for primary and secondary outcomes, including harms	p.12-13
Methods	21b	Who is included in each analysis; which group	p.13
Methods	21c	How missing data were handled	p.13
Methods	21d	Additional analyses (subgroup/sensitivity), pre vs post hoc	p.12-13
Results	22a	Participant flow (numbers randomised, received, analysed)	p.13+Fig 1
Results	22b	Losses and exclusions after randomisation, with reasons	p.13
Results	23a	Recruitment and follow-up dates	p.13
Results	23b	Why trial ended/stopped (if relevant)	None
Results	24a	Intervention/comparator as actually delivered (fidelity, adherence)	p.13-14
Results	24b	Concomitant care for each group	p.8-10

Results	25	Baseline data table	p.14+Table 1
Results	26	Outcomes for each group, effect size with precision; for binary, absolute & relative	p. 14-15;Tables 2-4; Figs 2-3 Supplemantary Table A B
Results	27	All harms/unintended effects	p.15+Table 3
Results	28	Ancillary analyses (subgroup/sensitivity), pre vs post hoc	p.15+Table 4
Discussion	29	Interpretation consistent with results & other evidence	p.16-19
Discussion	30	Limitations (bias, imprecision, generalisability, multiplicity)	p.18-19
Other	-	Ethical approval	p.6-7 and p.20
Other	-	Consent	p.7and p.20
Other	-	Registration UIN (re-stated)	p.7 and p.20-21
Other	-	Author contribution	p.20
Other	-	Provenance and peer review	p.21
