# Supplementary material for: Liposomal bupivacaine versus ropivacaine for surgical site infiltration in lumbar fusion: a prospective randomized controlled trial
Source: Ann Med. 2026 Jul 26;58(1):2704247. doi: 10.1080/07853890.2026.2704247 (PMC13410536; doi:10.1080/07853890.2026.2704247)
Supplement: Supplementary_Table_B.docx [file IANN_A_2704247_SM4502.docx]

**Supplementary Table B.** Descriptive raw postoperative NRS pain scores by time point

|  | LB group (n=101) | R group (n=101) |
| --- | --- | --- |
| Pain scores at rest |  |  |
| Postoperative 4 h | 1 (1, 2) | 2 (0, 2) |
| Postoperative 8 h | 2 (1, 2) | 2 (1, 2) |
| Postoperative 24 h | 1 (0, 1) | 1 (0, 2) |
| Postoperative 48 h | 1 (0, 1) | 1 (0, 2) |
| Postoperative 72 h | 0 (0, 1) | 1 (0, 1) |
| Pain scores with movement |  |  |
| Postoperative 4 h | 3 (1, 4) | 3 (2, 4) |
| Postoperative 8 h | 3 (2, 3) | 3 (2, 4) |
| Postoperative 24 h | 3 (2, 4) | 4 (3, 5) |
| Postoperative 48 h | 2 (2, 3) | 3 (2, 4) |
| Postoperative 72 h | 2 (1, 3) | 2 (2, 3) |

Raw time-point-specific values are provided for descriptive purposes only; no pointwise inferential testing was performed.

Data are median (IQR).

LB, liposomal bupivacaine; R, ropivacaine.
