# Supplementary material for: Liposomal bupivacaine versus ropivacaine for surgical site infiltration in lumbar fusion: a prospective randomized controlled trial
Source: Ann Med. 2026 Jul 26;58(1):2704247. doi: 10.1080/07853890.2026.2704247 (PMC13410536; doi:10.1080/07853890.2026.2704247)
Supplement: Supplementary Table A.docx [file IANN_A_2704247_SM4500.docx]

**Supplementary Table A.** Supportive model-based marginal mean postoperative NRS pain scores from the untransformed repeated-measures mixed-effects model

|  | LB group (n=101) | R group (n=101) | *P* |
| --- | --- | --- | --- |
| Pain scores at rest |  |  |  |
| Postoperative 4 h | 1.33 (1.13, 1.52) | 1.55 (1.35, 1.74) | 0.765 |
| Postoperative 8 h | 1.59 (1.40, 1.79) | 1.75 (1.60, 1.95) | 1.000 |
| Postoperative 24 h | 0.90 (0.71, 1.10) | 1.23 (1.03, 1.42) | 0.395 |
| Postoperative 48 h | 0.72 (0.53, 0.92) | 1.04 (0.85, 1.23) | 0.420 |
| Postoperative 72 h | 0.50 (0.30, 0.69) | 0.74 (0.55, 0.94) | 0.650 |
| Pain scores with movement |  |  |  |
| Postoperative 4 h | 2.72 (2.44, 3.00) | 2.92 (2.64, 3.20) | 1.000 |
| Postoperative 8 h | 2.69 (2.41, 2.97) | 3.00 (2.72, 3.28) | 0.645 |
| Postoperative 24 h | 2.91 (2.63, 3.19) | 3.57 (3.29, 3.85) | 0.065 |
| Postoperative 48 h | 2.55 (2.27, 2.84) | 3.30 (3.02, 3.58) | 0.055 |
| Postoperative 72 h | 2.20 (1.92, 2.48) | 2.50 (2.22, 2.78) | 0.710 |

Data are expressed as marginal means with 95% confidence intervals, estimated from the untransformed repeated-measures mixed-effects model. *P* values are Bonferroni-adjusted between-group pairwise comparisons at each postoperative time point from this untransformed model.

LB, liposomal bupivacaine; R, ropivacaine.
